# Naphthalene-2,3-diylbis[(2-thien­yl)methanone]

**DOI:** 10.1107/S1600536808038701

**Published:** 2008-11-26

**Authors:** S. Thenmozhi, A. SubbiahPandi, S. Ranjith, J. Arul Clement, A. K. MohanaKrishnan

**Affiliations:** a Department of Physics, Presidency College (Autonomous), Chennai 600 005, India; b Department of Organic Chemistry, University of Madras, Guindy Campus, Chennai 600 025, India

## Abstract

The asymmetric unit of the title compound, C_20_H_12_O_2_S_2_, contains two crystallographically independent mol­ecules which differ in the orientations of thienylmethanone units with respect to the naphthalene ring system [dihedral angles of 65.30 (11) and 50.94 (11)° in one molecule, 41.94 (12) and 69.61 (13)° in the other]. The crystal structure is stabilized by C—H⋯O and C—H⋯π inter­actions.

## Related literature

For a related structure, see: Labat & Halfpenny (2005 ▶). For general background, see: Pellis & West (1968 ▶); Cohen *et al.* (1977 ▶); Csaszar & Morvay (1983 ▶); Lakshmi *et al.* (1985 ▶); EI-Maghraby *et al.* (1984 ▶); Dzhurayev *et al.* (1992 ▶); Gewald *et al.* (1996 ▶); Jones *et al.* (1984 ▶); Palani *et al.* (2006 ▶).
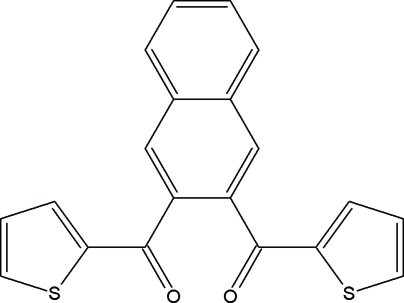


## Experimental

### 

#### Crystal data

C_20_H_12_O_2_S_2_
*M* *_r_* = 348.42
Monoclinic, 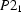

*a* = 9.7638 (3) Å
*b* = 11.1418 (4) Å
*c* = 15.4496 (6) Å
β = 90.266 (1)°
*V* = 1680.69 (10) Å^3^
*Z* = 4
Mo *K*α radiation
μ = 0.33 mm^−1^
*T* = 293 (2) K
0.21 × 0.19 × 0.16 mm

#### Data collection

Bruker APEXII CCD area-detector diffractometer
Absorption correction: multi-scan (*SADABS*; Sheldrick, 1996 ▶) *T*_min_ = 0.800, *T*_max_ = 0.950
21289 measured reflections
8580 independent reflections
6591 reflections with *I* > 2σ(*I*)
*R*_int_ = 0.024

#### Refinement

*R*[*F* ^2^ > 2σ(*F* ^2^)] = 0.044
*wR*(*F* ^2^) = 0.112
*S* = 1.01
8580 reflections
433 parameters
1 restraint
H-atom parameters constrained
Δρ_max_ = 0.44 e Å^−3^
Δρ_min_ = −0.32 e Å^−3^
Absolute structure: Flack (1983 ▶), 3970 Friedel pairs
Flack parameter: 0.02 (5)

### 

Data collection: *APEX2* (Bruker, 2004 ▶); cell refinement: *SAINT* (Bruker, 2004 ▶); data reduction: *SAINT*; program(s) used to solve structure: *SHELXS97* (Sheldrick, 2008 ▶); program(s) used to refine structure: *SHELXL97* (Sheldrick, 2008 ▶); molecular graphics: *ORTEP-3* (Farrugia, 1997 ▶); software used to prepare material for publication: *SHELXL97* and *PLATON* (Spek, 2003 ▶).

## Supplementary Material

Crystal structure: contains datablocks global, I. DOI: 10.1107/S1600536808038701/ci2690sup1.cif

Structure factors: contains datablocks I. DOI: 10.1107/S1600536808038701/ci2690Isup2.hkl

Additional supplementary materials:  crystallographic information; 3D view; checkCIF report

## Figures and Tables

**Table 1:** Hydrogen-bond geometry (Å, °)

*D*—H⋯*A*	*D*—H	H⋯*A*	*D*⋯*A*	*D*—H⋯*A*
C4—H4⋯O2^i^	0.93	2.53	3.407 (3)	158
C13—H13⋯*Cg*1^ii^	0.93	2.86	3.737 (2)	157

Symmetry codes: (i) 
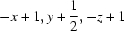
; (ii) 
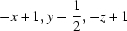
. *Cg*1 is the S2/C17–C20 ring centroid.

**Fig. 1.**
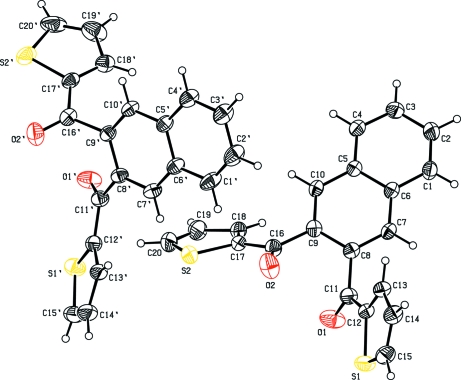
The asymmetric unit of the title compound. Displacement ellipsoids are drawn at the 30% probability level.

**Fig. 2.**
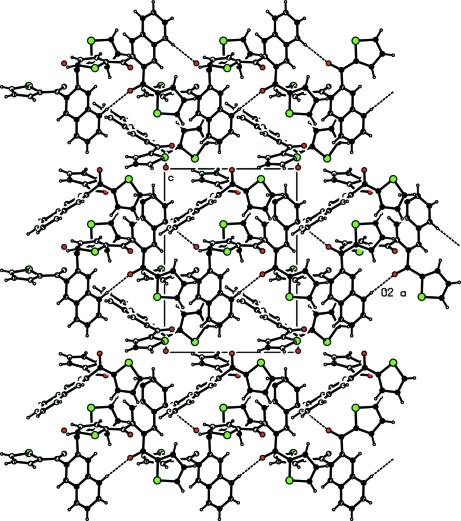
The crystal packing of the title compound, viewed down the b axis. Hydrogen bonds are shown as dashed lines.

## supplementary crystallographic information

### Comment

Sulfur containing Schiff bases (Pellis & West, 1968; Cohen *et al.*, 1977; Csaszar & Morvay,1983; Lakshmi *et al.*, 1985), and their thiophene derivatives (EI-Maghraby *et al.*, 1984; Dzhurayev *et al.*, 1992), possess pharmacological activities such as anti-bacterial, anti-cancer, anti-inflammatory and anti-toxic properties (Gewald *et al.*, 1996). Benzo(*b*)thiophene analogs have been shown to possess interesting estrogenic and antiestrogenic effects. Some of these compounds inhibit estradiol with greater potency than tamoxifen, and inhibition of the growth of DMBA induced mammary tumors by such compounds has been reported (Jones *et al.*, 1984). Some of the thiophene derivatives were screened against gram-positive, gram-negative bacteria and have shown promising anti-bacterial activity (Palani *et al.*, 2006). In view of this biological importance, the crystal structure of the title compound has been determined and the results are presented here.

The asymmetric unit of the title compound contains two crystallographically independent molecules (Fig. 1). The corresponding bond lengths and angles of the two molecules agree with each other, and are comparable to those observed in the structure of 1,5-bis(3-thienyloxy)-3-oxapentane (Labat & Halfpenny, 2005). The two independent molecules differ in the orientations of thienylmethanone units with respect to the naphthalene ring system. The S1/C12-C15 and S2/C17-C20 rings form dihedral angles of 65.30 (11)° and 50.94 (11)°, respectively, with the C1-C10 naphthalene ring system, whereas, the S1'/C12'-C15' and S2'/C17'-C20' rings form dihedral angles of 41.94 (12)° and 69.61 (13)°, respectively, with the C1'-C10' naphthalene ring system.

The crystal structure is stabilized by C–H···O and C–H···π interactions (Table 1 and Fig.2).

### Experimental

A mixture of phthalaldehyde (1 g, 7.46 mmol) and 1,4-di(thiophene-2-yl)butane- 1,4-diane (1.86 g 7.46 mmol) was dissolved in ethanol and tertiary-butane oxide (2.08 g, 18.6 mmol), and then allowed to stir for 4 h at room temperature to get the product. The crude product was filtered and then recrystallized in chloroform. Single crystals suitable for X-ray diffraction were obtained by slow evaporation of a chloroform solution at room temperature.

### Refinement

All H atoms were fixed geometrically and allowed to ride on their parent C atoms, with C-H = 0.93 Å and *U*_iso_(H) = 1.2*U*_eq_(C).

### Crystal data

**Table:**

C_20_H_12_O_2_S_2_	*F*_000_ = 720
*M**_r_* = 348.42	*D*_x_ = 1.377 Mg m^−^^3^
Monoclinic, *P*2_1_	Mo *K*α radiation λ = 0.71073 Å
Hall symbol: P 2yb	Cell parameters from 8580 reflections
*a* = 9.7638 (3) Å	θ = 1.3–28.9º
*b* = 11.1418 (4) Å	µ = 0.33 mm^−^^1^
*c* = 15.4496 (6) Å	*T* = 293 (2) K
β = 90.266 (1)º	Block, colourless
*V* = 1680.69 (10) Å^3^	0.21 × 0.19 × 0.16 mm
*Z* = 4	

### Data collection

**Table:**

Bruker APEXII CCD area-detector diffractometer	8580 independent reflections
Radiation source: fine-focus sealed tube	6591 reflections with *I* > 2σ(*I*)
Monochromator: graphite	*R*_int_ = 0.024
*T* = 293(2) K	θ_max_ = 28.9º
ω scans	θ_min_ = 1.3º
Absorption correction: multi-scan(SADABS; Sheldrick, 1996)	*h* = −13→13
*T*_min_ = 0.800, *T*_max_ = 0.950	*k* = −15→14
21289 measured reflections	*l* = −20→17

### Refinement

**Table:**

Refinement on *F*^2^	Hydrogen site location: inferred from neighbouring sites
Least-squares matrix: full	H-atom parameters constrained
*R*[*F*^2^ > 2σ(*F*^2^)] = 0.044	*w* = 1/[σ^2^(*F*_o_^2^) + (0.0499*P*)^2^ + 0.3545*P*] where *P* = (*F*_o_^2^ + 2*F*_c_^2^)/3
*wR*(*F*^2^) = 0.112	(Δ/σ)_max_ = 0.001
*S* = 1.01	Δρ_max_ = 0.44 e Å^−^^3^
8580 reflections	Δρ_min_ = −0.32 e Å^−^^3^
433 parameters	Extinction correction: none
1 restraint	Absolute structure: Flack (1983), 3970 Friedel pairs
Primary atom site location: structure-invariant direct methods	Flack parameter: 0.02 (5)
Secondary atom site location: difference Fourier map	

### Special details

**Table:**

Geometry. All e.s.d.'s (except the e.s.d. in the dihedral angle between two l.s. planes) are estimated using the full covariance matrix. The cell e.s.d.'s are taken into account individually in the estimation of e.s.d.'s in distances, angles and torsion angles; correlations between e.s.d.'s in cell parameters are only used when they are defined by crystal symmetry. An approximate (isotropic) treatment of cell e.s.d.'s is used for estimating e.s.d.'s involving l.s. planes.
Refinement. Refinement of *F*^2^ against ALL reflections. The weighted *R*-factor *wR* and goodness of fit *S* are based on *F*^2^, conventional *R*-factors *R* are based on *F*, with *F* set to zero for negative *F*^2^. The threshold expression of *F*^2^ > σ(*F*^2^) is used only for calculating *R*-factors(gt) *etc*. and is not relevant to the choice of reflections for refinement. *R*-factors based on *F*^2^ are statistically about twice as large as those based on *F*, and *R*- factors based on ALL data will be even larger.

### Fractional atomic coordinates and isotropic or equivalent isotropic displacement parameters (Å^2^)

**Table:**

	*x*	*y*	*z*	*U*_iso_*/*U*_eq_	
S1	0.06339 (8)	−0.02637 (6)	0.45118 (6)	0.0657 (2)	
S2	0.33739 (8)	0.44467 (8)	0.69777 (5)	0.0644 (2)	
O1	0.0949 (2)	0.24092 (19)	0.44816 (17)	0.0822 (7)	
O2	0.3446 (3)	0.24071 (18)	0.57466 (14)	0.0815 (7)	
C1	0.4852 (3)	0.3351 (2)	0.18008 (17)	0.0542 (6)	
H1	0.4468	0.2785	0.1429	0.065*	
C2	0.5757 (3)	0.4164 (3)	0.14849 (18)	0.0609 (7)	
H2	0.5984	0.4148	0.0901	0.073*	
C3	0.6350 (3)	0.5021 (3)	0.20256 (18)	0.0571 (6)	
H3	0.6966	0.5574	0.1800	0.069*	
C4	0.6032 (2)	0.5053 (2)	0.28836 (17)	0.0488 (6)	
H4	0.6430	0.5631	0.3239	0.059*	
C5	0.5102 (2)	0.42129 (19)	0.32380 (15)	0.0398 (5)	
C6	0.4485 (2)	0.3350 (2)	0.26773 (16)	0.0421 (5)	
C7	0.3516 (2)	0.2545 (2)	0.30259 (16)	0.0451 (5)	
H7	0.3127	0.1966	0.2668	0.054*	
C8	0.3135 (2)	0.25942 (19)	0.38716 (16)	0.0434 (5)	
C9	0.3768 (2)	0.3442 (2)	0.44373 (16)	0.0442 (5)	
C10	0.4727 (2)	0.4227 (2)	0.41136 (15)	0.0428 (5)	
H10	0.5137	0.4779	0.4484	0.051*	
C11	0.1943 (3)	0.1879 (2)	0.42024 (19)	0.0508 (6)	
C12	0.2010 (2)	0.0578 (2)	0.41656 (16)	0.0448 (5)	
C13	0.3071 (2)	−0.0134 (2)	0.39437 (17)	0.0484 (6)	
H13	0.3903	0.0156	0.3741	0.058*	
C14	0.2781 (3)	−0.1368 (2)	0.4053 (2)	0.0608 (7)	
H14	0.3397	−0.1980	0.3929	0.073*	
C15	0.1515 (3)	−0.1553 (3)	0.4355 (2)	0.0653 (8)	
H15	0.1158	−0.2310	0.4467	0.078*	
C16	0.3484 (3)	0.3376 (2)	0.53847 (17)	0.0506 (6)	
C17	0.3315 (2)	0.4492 (2)	0.58672 (15)	0.0456 (5)	
C18	0.3095 (3)	0.5646 (2)	0.55646 (19)	0.0521 (6)	
H18	0.3026	0.5854	0.4983	0.062*	
C19	0.2991 (3)	0.6466 (3)	0.6262 (2)	0.0696 (8)	
H19	0.2841	0.7284	0.6186	0.084*	
C20	0.3127 (3)	0.5957 (3)	0.7037 (2)	0.0722 (9)	
H20	0.3091	0.6381	0.7555	0.087*	
S1'	0.36488 (7)	0.50071 (8)	0.93775 (5)	0.0670 (2)	
S2'	1.03585 (9)	0.72925 (8)	0.94344 (7)	0.0795 (3)	
O1'	0.6344 (2)	0.55500 (17)	0.87437 (16)	0.0701 (6)	
O2'	0.8753 (3)	0.5098 (2)	0.99329 (14)	0.0745 (6)	
C1'	0.7926 (3)	0.0907 (2)	0.7211 (2)	0.0694 (9)	
H1'	0.7053	0.0575	0.7197	0.083*	
C2'	0.8959 (4)	0.0362 (2)	0.6773 (2)	0.0730 (9)	
H2'	0.8777	−0.0319	0.6445	0.088*	
C3'	1.0280 (3)	0.0812 (2)	0.6812 (2)	0.0677 (8)	
H3'	1.0981	0.0421	0.6521	0.081*	
C4'	1.0559 (3)	0.1833 (2)	0.7278 (2)	0.0597 (7)	
H4'	1.1449	0.2130	0.7300	0.072*	
C5'	0.9510 (3)	0.2433 (2)	0.77216 (17)	0.0469 (6)	
C6'	0.8160 (3)	0.1970 (2)	0.76870 (18)	0.0498 (6)	
C7'	0.7096 (3)	0.2617 (2)	0.80915 (18)	0.0526 (6)	
H7'	0.6207	0.2320	0.8061	0.063*	
C8'	0.7328 (2)	0.3666 (2)	0.85265 (17)	0.0450 (5)	
C9'	0.8698 (2)	0.40998 (19)	0.85839 (16)	0.0433 (5)	
C10'	0.9736 (3)	0.3506 (2)	0.81877 (18)	0.0491 (6)	
H10'	1.0620	0.3814	0.8224	0.059*	
C11'	0.6203 (3)	0.4464 (2)	0.88117 (17)	0.0490 (6)	
C12'	0.4927 (3)	0.3989 (2)	0.91630 (17)	0.0484 (6)	
C13'	0.4546 (3)	0.2823 (3)	0.93971 (18)	0.0561 (7)	
H13'	0.5098	0.2148	0.9340	0.067*	
C14'	0.3192 (3)	0.2820 (3)	0.9735 (2)	0.0713 (9)	
H14'	0.2752	0.2126	0.9921	0.086*	
C15'	0.2605 (3)	0.3915 (3)	0.9763 (2)	0.0723 (9)	
H15'	0.1727	0.4056	0.9970	0.087*	
C16'	0.9038 (3)	0.5142 (2)	0.91669 (18)	0.0495 (6)	
C17'	0.9723 (2)	0.6167 (2)	0.87875 (19)	0.0511 (6)	
C18'	0.9843 (3)	0.6463 (2)	0.7926 (2)	0.0577 (7)	
H18'	0.9581	0.5964	0.7472	0.069*	
C19'	1.0413 (4)	0.7619 (3)	0.7816 (3)	0.0902 (12)	
H19'	1.0548	0.7975	0.7278	0.108*	
C20'	1.0736 (4)	0.8144 (3)	0.8562 (3)	0.0952 (14)	
H20'	1.1130	0.8902	0.8600	0.114*	

### Atomic displacement parameters (Å^2^)

**Table:**

	*U*^11^	*U*^22^	*U*^33^	*U*^12^	*U*^13^	*U*^23^
S1	0.0555 (4)	0.0583 (4)	0.0835 (5)	−0.0129 (3)	0.0201 (4)	−0.0068 (4)
S2	0.0706 (5)	0.0755 (5)	0.0471 (4)	−0.0149 (4)	0.0070 (3)	−0.0040 (4)
O1	0.0665 (13)	0.0542 (12)	0.126 (2)	0.0072 (10)	0.0366 (13)	−0.0069 (13)
O2	0.135 (2)	0.0491 (11)	0.0603 (13)	0.0066 (12)	0.0098 (13)	0.0124 (10)
C1	0.0642 (17)	0.0556 (15)	0.0428 (15)	−0.0048 (12)	−0.0067 (12)	−0.0048 (12)
C2	0.0709 (19)	0.0683 (18)	0.0437 (15)	−0.0089 (15)	0.0013 (14)	0.0006 (13)
C3	0.0580 (15)	0.0573 (15)	0.0562 (16)	−0.0080 (13)	0.0039 (13)	0.0054 (13)
C4	0.0466 (13)	0.0469 (12)	0.0527 (15)	−0.0022 (11)	−0.0047 (11)	−0.0053 (11)
C5	0.0392 (11)	0.0353 (10)	0.0448 (13)	0.0058 (9)	−0.0047 (10)	−0.0025 (9)
C6	0.0440 (12)	0.0368 (10)	0.0455 (14)	0.0039 (9)	−0.0051 (10)	−0.0031 (9)
C7	0.0466 (13)	0.0396 (11)	0.0491 (15)	−0.0017 (9)	−0.0061 (11)	−0.0072 (10)
C8	0.0438 (12)	0.0333 (10)	0.0530 (15)	0.0032 (9)	−0.0011 (11)	−0.0019 (10)
C9	0.0478 (13)	0.0389 (11)	0.0460 (14)	0.0070 (10)	−0.0008 (11)	−0.0008 (10)
C10	0.0462 (12)	0.0382 (11)	0.0439 (13)	0.0019 (9)	−0.0068 (10)	−0.0060 (10)
C11	0.0481 (14)	0.0435 (12)	0.0610 (17)	0.0015 (10)	0.0054 (12)	−0.0060 (11)
C12	0.0433 (13)	0.0437 (11)	0.0474 (14)	−0.0074 (10)	−0.0008 (11)	−0.0020 (10)
C13	0.0421 (12)	0.0449 (12)	0.0582 (16)	−0.0006 (10)	−0.0013 (11)	0.0002 (11)
C14	0.0656 (18)	0.0427 (13)	0.074 (2)	−0.0007 (12)	−0.0019 (15)	−0.0039 (13)
C15	0.074 (2)	0.0439 (13)	0.078 (2)	−0.0143 (13)	0.0014 (16)	−0.0052 (14)
C16	0.0563 (15)	0.0479 (13)	0.0477 (15)	−0.0002 (11)	0.0008 (12)	0.0014 (11)
C17	0.0433 (12)	0.0505 (13)	0.0429 (13)	−0.0061 (10)	0.0021 (10)	−0.0061 (11)
C18	0.0522 (15)	0.0505 (13)	0.0536 (16)	−0.0017 (11)	0.0036 (12)	−0.0111 (12)
C19	0.082 (2)	0.0509 (15)	0.076 (2)	−0.0088 (14)	0.0077 (17)	−0.0145 (15)
C20	0.085 (2)	0.0714 (19)	0.061 (2)	−0.0177 (17)	0.0105 (17)	−0.0213 (16)
S1'	0.0518 (4)	0.0751 (5)	0.0741 (5)	0.0064 (4)	0.0049 (3)	0.0109 (4)
S2'	0.0642 (5)	0.0705 (5)	0.1039 (7)	−0.0178 (4)	0.0145 (4)	−0.0433 (5)
O1'	0.0608 (12)	0.0444 (10)	0.1051 (18)	0.0024 (8)	0.0171 (12)	0.0143 (10)
O2'	0.1087 (17)	0.0605 (11)	0.0544 (13)	−0.0031 (12)	0.0069 (12)	−0.0051 (10)
C1'	0.0614 (17)	0.0451 (14)	0.102 (3)	−0.0143 (13)	−0.0104 (17)	−0.0143 (15)
C2'	0.077 (2)	0.0430 (15)	0.099 (3)	−0.0012 (13)	−0.0106 (18)	−0.0220 (15)
C3'	0.0627 (18)	0.0486 (15)	0.092 (2)	0.0101 (13)	−0.0075 (16)	−0.0179 (15)
C4'	0.0483 (14)	0.0480 (14)	0.083 (2)	0.0035 (11)	−0.0085 (14)	−0.0124 (14)
C5'	0.0477 (13)	0.0370 (11)	0.0559 (15)	−0.0005 (10)	−0.0089 (11)	−0.0013 (11)
C6'	0.0494 (13)	0.0375 (11)	0.0624 (16)	−0.0048 (10)	−0.0072 (12)	−0.0010 (11)
C7'	0.0459 (14)	0.0474 (13)	0.0645 (17)	−0.0124 (11)	−0.0019 (12)	0.0012 (12)
C8'	0.0424 (12)	0.0398 (12)	0.0528 (15)	−0.0050 (9)	−0.0026 (11)	0.0064 (10)
C9'	0.0453 (13)	0.0356 (11)	0.0489 (14)	−0.0034 (9)	−0.0055 (11)	0.0015 (9)
C10'	0.0417 (13)	0.0427 (12)	0.0629 (17)	−0.0068 (10)	−0.0077 (12)	−0.0056 (12)
C11'	0.0474 (13)	0.0449 (12)	0.0547 (15)	−0.0022 (10)	−0.0015 (11)	0.0052 (11)
C12'	0.0455 (13)	0.0548 (13)	0.0447 (14)	−0.0021 (10)	−0.0032 (11)	0.0053 (11)
C13'	0.0575 (16)	0.0607 (15)	0.0502 (16)	−0.0155 (12)	0.0022 (13)	0.0066 (13)
C14'	0.0674 (19)	0.080 (2)	0.067 (2)	−0.0244 (17)	0.0051 (16)	0.0108 (17)
C15'	0.0505 (16)	0.097 (2)	0.070 (2)	−0.0097 (16)	0.0056 (15)	0.0123 (18)
C16'	0.0467 (13)	0.0436 (12)	0.0580 (17)	0.0035 (10)	−0.0070 (12)	−0.0038 (12)
C17'	0.0399 (13)	0.0418 (11)	0.0717 (18)	−0.0020 (10)	0.0014 (12)	−0.0172 (12)
C18'	0.0589 (16)	0.0415 (13)	0.073 (2)	−0.0023 (11)	0.0052 (14)	−0.0020 (12)
C19'	0.102 (3)	0.0523 (18)	0.116 (3)	−0.0066 (17)	0.041 (2)	0.0019 (19)
C20'	0.080 (2)	0.0568 (18)	0.150 (4)	−0.0257 (17)	0.051 (2)	−0.027 (2)

### Geometric parameters (Å, °)

**Table:**

S1—C15	1.692 (3)	S1'—C15'	1.697 (3)
S1—C12	1.726 (2)	S1'—C12'	1.720 (3)
S2—C20	1.702 (4)	S2'—C20'	1.690 (4)
S2—C17	1.717 (2)	S2'—C17'	1.718 (2)
O1—C11	1.216 (3)	O1'—C11'	1.222 (3)
O2—C16	1.216 (3)	O2'—C16'	1.218 (3)
C1—C2	1.358 (4)	C1'—C2'	1.361 (5)
C1—C6	1.402 (4)	C1'—C6'	1.412 (4)
C1—H1	0.93	C1'—H1'	0.93
C2—C3	1.393 (4)	C2'—C3'	1.384 (4)
C2—H2	0.93	C2'—H2'	0.93
C3—C4	1.363 (4)	C3'—C4'	1.373 (4)
C3—H3	0.93	C3'—H3'	0.93
C4—C5	1.416 (3)	C4'—C5'	1.404 (4)
C4—H4	0.93	C4'—H4'	0.93
C5—C10	1.403 (3)	C5'—C10'	1.413 (3)
C5—C6	1.426 (3)	C5'—C6'	1.416 (3)
C6—C7	1.412 (3)	C6'—C7'	1.412 (4)
C7—C8	1.361 (4)	C7'—C8'	1.366 (3)
C7—H7	0.93	C7'—H7'	0.93
C8—C9	1.425 (3)	C8'—C9'	1.424 (3)
C8—C11	1.503 (4)	C8'—C11'	1.482 (4)
C9—C10	1.377 (3)	C9'—C10'	1.359 (4)
C9—C16	1.493 (4)	C9'—C16'	1.506 (3)
C10—H10	0.93	C10'—H10'	0.93
C11—C12	1.452 (3)	C11'—C12'	1.461 (4)
C12—C13	1.350 (3)	C12'—C13'	1.398 (4)
C13—C14	1.414 (4)	C13'—C14'	1.424 (4)
C13—H13	0.93	C13'—H13'	0.93
C14—C15	1.339 (4)	C14'—C15'	1.348 (5)
C14—H14	0.93	C14'—H14'	0.93
C15—H15	0.93	C15'—H15'	0.93
C16—C17	1.459 (4)	C16'—C17'	1.448 (4)
C17—C18	1.385 (4)	C17'—C18'	1.377 (4)
C18—C19	1.418 (4)	C18'—C19'	1.414 (4)
C18—H18	0.93	C18'—H18'	0.93
C19—C20	1.330 (5)	C19'—C20'	1.329 (6)
C19—H19	0.93	C19'—H19'	0.93
C20—H20	0.93	C20'—H20'	0.93
			
C15—S1—C12	91.10 (14)	C15'—S1'—C12'	91.82 (16)
C20—S2—C17	91.19 (15)	C20'—S2'—C17'	91.46 (17)
C2—C1—C6	121.1 (2)	C2'—C1'—C6'	121.0 (3)
C2—C1—H1	119.4	C2'—C1'—H1'	119.5
C6—C1—H1	119.4	C6'—C1'—H1'	119.5
C1—C2—C3	120.7 (3)	C1'—C2'—C3'	120.6 (3)
C1—C2—H2	119.6	C1'—C2'—H2'	119.7
C3—C2—H2	119.6	C3'—C2'—H2'	119.7
C4—C3—C2	120.4 (3)	C4'—C3'—C2'	120.4 (3)
C4—C3—H3	119.8	C4'—C3'—H3'	119.8
C2—C3—H3	119.8	C2'—C3'—H3'	119.8
C3—C4—C5	120.5 (2)	C3'—C4'—C5'	120.4 (3)
C3—C4—H4	119.7	C3'—C4'—H4'	119.8
C5—C4—H4	119.7	C5'—C4'—H4'	119.8
C10—C5—C4	122.4 (2)	C4'—C5'—C10'	122.6 (2)
C10—C5—C6	118.8 (2)	C4'—C5'—C6'	119.3 (2)
C4—C5—C6	118.7 (2)	C10'—C5'—C6'	118.1 (2)
C1—C6—C7	122.9 (2)	C1'—C6'—C7'	122.8 (2)
C1—C6—C5	118.5 (2)	C1'—C6'—C5'	118.2 (3)
C7—C6—C5	118.6 (2)	C7'—C6'—C5'	118.9 (2)
C8—C7—C6	121.8 (2)	C8'—C7'—C6'	122.3 (2)
C8—C7—H7	119.1	C8'—C7'—H7'	118.9
C6—C7—H7	119.1	C6'—C7'—H7'	118.9
C7—C8—C9	119.7 (2)	C7'—C8'—C9'	118.3 (2)
C7—C8—C11	121.4 (2)	C7'—C8'—C11'	122.5 (2)
C9—C8—C11	118.5 (2)	C9'—C8'—C11'	118.4 (2)
C10—C9—C8	119.4 (2)	C10'—C9'—C8'	120.6 (2)
C10—C9—C16	121.1 (2)	C10'—C9'—C16'	118.8 (2)
C8—C9—C16	119.1 (2)	C8'—C9'—C16'	120.2 (2)
C9—C10—C5	121.6 (2)	C9'—C10'—C5'	121.8 (2)
C9—C10—H10	119.2	C9'—C10'—H10'	119.1
C5—C10—H10	119.2	C5'—C10'—H10'	119.1
O1—C11—C12	122.4 (3)	O1'—C11'—C12'	119.2 (2)
O1—C11—C8	118.9 (2)	O1'—C11'—C8'	119.0 (2)
C12—C11—C8	118.7 (2)	C12'—C11'—C8'	121.8 (2)
C13—C12—C11	129.1 (2)	C13'—C12'—C11'	131.4 (3)
C13—C12—S1	111.06 (19)	C13'—C12'—S1'	111.6 (2)
C11—C12—S1	119.6 (2)	C11'—C12'—S1'	116.94 (19)
C12—C13—C14	112.7 (2)	C12'—C13'—C14'	110.2 (3)
C12—C13—H13	123.6	C12'—C13'—H13'	124.9
C14—C13—H13	123.6	C14'—C13'—H13'	124.9
C15—C14—C13	112.1 (3)	C15'—C14'—C13'	113.9 (3)
C15—C14—H14	123.9	C15'—C14'—H14'	123.1
C13—C14—H14	123.9	C13'—C14'—H14'	123.1
C14—C15—S1	113.0 (2)	C14'—C15'—S1'	112.5 (3)
C14—C15—H15	123.5	C14'—C15'—H15'	123.8
S1—C15—H15	123.5	S1'—C15'—H15'	123.8
O2—C16—C17	121.2 (2)	O2'—C16'—C17'	122.2 (2)
O2—C16—C9	120.0 (2)	O2'—C16'—C9'	120.0 (2)
C17—C16—C9	118.7 (2)	C17'—C16'—C9'	117.8 (2)
C18—C17—C16	129.5 (2)	C18'—C17'—C16'	128.5 (2)
C18—C17—S2	111.64 (19)	C18'—C17'—S2'	110.8 (2)
C16—C17—S2	118.83 (19)	C16'—C17'—S2'	120.4 (2)
C17—C18—C19	110.7 (3)	C17'—C18'—C19'	111.8 (3)
C17—C18—H18	124.6	C17'—C18'—H18'	124.1
C19—C18—H18	124.6	C19'—C18'—H18'	124.1
C20—C19—C18	113.7 (3)	C20'—C19'—C18'	112.8 (4)
C20—C19—H19	123.2	C20'—C19'—H19'	123.6
C18—C19—H19	123.2	C18'—C19'—H19'	123.6
C19—C20—S2	112.8 (2)	C19'—C20'—S2'	113.2 (3)
C19—C20—H20	123.6	C19'—C20'—H20'	123.4
S2—C20—H20	123.6	S2'—C20'—H20'	123.4
			
C6—C1—C2—C3	0.1 (4)	C6'—C1'—C2'—C3'	2.6 (6)
C1—C2—C3—C4	0.2 (4)	C1'—C2'—C3'—C4'	−1.5 (6)
C2—C3—C4—C5	0.3 (4)	C2'—C3'—C4'—C5'	0.1 (5)
C3—C4—C5—C10	−178.8 (2)	C3'—C4'—C5'—C10'	−178.0 (3)
C3—C4—C5—C6	−1.2 (3)	C3'—C4'—C5'—C6'	0.3 (4)
C2—C1—C6—C7	177.9 (3)	C2'—C1'—C6'—C7'	174.7 (3)
C2—C1—C6—C5	−1.0 (4)	C2'—C1'—C6'—C5'	−2.1 (5)
C10—C5—C6—C1	179.2 (2)	C4'—C5'—C6'—C1'	0.7 (4)
C4—C5—C6—C1	1.5 (3)	C10'—C5'—C6'—C1'	179.1 (3)
C10—C5—C6—C7	0.3 (3)	C4'—C5'—C6'—C7'	−176.3 (2)
C4—C5—C6—C7	−177.5 (2)	C10'—C5'—C6'—C7'	2.1 (4)
C1—C6—C7—C8	−177.3 (2)	C1'—C6'—C7'—C8'	−177.8 (3)
C5—C6—C7—C8	1.6 (3)	C5'—C6'—C7'—C8'	−1.0 (4)
C6—C7—C8—C9	−2.8 (3)	C6'—C7'—C8'—C9'	−1.3 (4)
C6—C7—C8—C11	169.7 (2)	C6'—C7'—C8'—C11'	168.4 (2)
C7—C8—C9—C10	2.0 (3)	C7'—C8'—C9'—C10'	2.4 (4)
C11—C8—C9—C10	−170.7 (2)	C11'—C8'—C9'—C10'	−167.7 (2)
C7—C8—C9—C16	−171.7 (2)	C7'—C8'—C9'—C16'	−170.7 (2)
C11—C8—C9—C16	15.6 (3)	C11'—C8'—C9'—C16'	19.2 (3)
C8—C9—C10—C5	−0.1 (3)	C8'—C9'—C10'—C5'	−1.2 (4)
C16—C9—C10—C5	173.5 (2)	C16'—C9'—C10'—C5'	171.9 (2)
C4—C5—C10—C9	176.6 (2)	C4'—C5'—C10'—C9'	177.3 (3)
C6—C5—C10—C9	−1.0 (3)	C6'—C5'—C10'—C9'	−1.0 (4)
C7—C8—C11—O1	−116.2 (3)	C7'—C8'—C11'—O1'	−139.6 (3)
C9—C8—C11—O1	56.4 (4)	C9'—C8'—C11'—O1'	30.0 (4)
C7—C8—C11—C12	63.0 (3)	C7'—C8'—C11'—C12'	39.7 (4)
C9—C8—C11—C12	−124.5 (3)	C9'—C8'—C11'—C12'	−150.6 (2)
O1—C11—C12—C13	−173.9 (3)	O1'—C11'—C12'—C13'	−172.2 (3)
C8—C11—C12—C13	7.0 (4)	C8'—C11'—C12'—C13'	8.4 (4)
O1—C11—C12—S1	1.0 (4)	O1'—C11'—C12'—S1'	5.0 (4)
C8—C11—C12—S1	−178.11 (18)	C8'—C11'—C12'—S1'	−174.30 (19)
C15—S1—C12—C13	0.2 (2)	C15'—S1'—C12'—C13'	−0.6 (2)
C15—S1—C12—C11	−175.6 (2)	C15'—S1'—C12'—C11'	−178.4 (2)
C11—C12—C13—C14	175.2 (3)	C11'—C12'—C13'—C14'	178.2 (3)
S1—C12—C13—C14	−0.1 (3)	S1'—C12'—C13'—C14'	0.9 (3)
C12—C13—C14—C15	−0.2 (4)	C12'—C13'—C14'—C15'	−0.8 (4)
C13—C14—C15—S1	0.3 (4)	C13'—C14'—C15'—S1'	0.3 (4)
C12—S1—C15—C14	−0.3 (3)	C12'—S1'—C15'—C14'	0.1 (3)
C10—C9—C16—O2	−131.8 (3)	C10'—C9'—C16'—O2'	−117.6 (3)
C8—C9—C16—O2	41.8 (4)	C8'—C9'—C16'—O2'	55.6 (3)
C10—C9—C16—C17	45.5 (3)	C10'—C9'—C16'—C17'	61.4 (3)
C8—C9—C16—C17	−140.8 (2)	C8'—C9'—C16'—C17'	−125.4 (2)
O2—C16—C17—C18	−167.8 (3)	O2'—C16'—C17'—C18'	−164.4 (3)
C9—C16—C17—C18	14.9 (4)	C9'—C16'—C17'—C18'	16.6 (4)
O2—C16—C17—S2	11.8 (4)	O2'—C16'—C17'—S2'	8.2 (4)
C9—C16—C17—S2	−165.52 (19)	C9'—C16'—C17'—S2'	−170.75 (18)
C20—S2—C17—C18	−0.6 (2)	C20'—S2'—C17'—C18'	1.2 (2)
C20—S2—C17—C16	179.7 (2)	C20'—S2'—C17'—C16'	−172.6 (2)
C16—C17—C18—C19	−180.0 (3)	C16'—C17'—C18'—C19'	171.4 (3)
S2—C17—C18—C19	0.4 (3)	S2'—C17'—C18'—C19'	−1.8 (3)
C17—C18—C19—C20	0.1 (4)	C17'—C18'—C19'—C20'	1.6 (4)
C18—C19—C20—S2	−0.6 (4)	C18'—C19'—C20'—S2'	−0.7 (5)
C17—S2—C20—C19	0.7 (3)	C17'—S2'—C20'—C19'	−0.3 (3)

### Hydrogen-bond geometry (Å, °)

**Table:**

*D*—H···*A*	*D*—H	H···*A*	*D*···*A*	*D*—H···*A*
C4—H4···O2^i^	0.93	2.53	3.407 (3)	158
C13—H13···Cg1^ii^	0.93	2.86	3.737 (2)	157

Symmetry codes: (i) −*x*+1, *y*+1/2, −*z*+1; (ii) −*x*+1, *y*−1/2, −*z*+1.
